# Therapeutic applications of CRISPR/Cas9 mediated targeted gene editing in acute lymphoblastic leukemia: current perspectives, future challenges, and clinical implications

**DOI:** 10.3389/fphar.2023.1322937

**Published:** 2023-12-07

**Authors:** Alan Jhones Barbosa Assis, Brunna Letícia de Oliveira Santana, Ana Cristina Moura Gualberto, Fabio Pittella-Silva

**Affiliations:** Laboratory of Molecular Pathology of Cancer, Faculty of Health Sciences and Medicine, University of Brasília, Brasília, Brazil

**Keywords:** CRISPR/Cas9, gene editing, acute lymphoblastic leukemia, therapeutic strategies cancer treatment, current perspectives

## Abstract

Acute Lymphoblastic Leukemia (ALL) is the predominant hematological malignancy in pediatric populations, originating from B- or T-cell precursors within the bone marrow. The disease exhibits a high degree of heterogeneity, both at the molecular level and in terms of clinical presentation. A complex interplay between inherited and acquired genetic alterations contributes to disease pathogenesis, often resulting in the disruption of cellular functions integral to the leukemogenic process. The advent of CRISPR/Cas9 as a gene editing tool has revolutionized biological research, underscoring its potential to modify specific genomic loci implicated in cancer. Enhanced understanding of molecular alterations in ALL has facilitated significant advancements in therapeutic strategies. In this review, we scrutinize the application of CRISPR/Cas9 as a tool for identifying genetic targets to improve therapy, circumvent drug resistance, and facilitate CAR-T cell-based immunotherapy. Additionally, we discuss the challenges and future prospects of CRISPR/Cas9 applications in ALL.

## 1 Introduction

Acute lymphoblastic leukemia (ALL) is a type of hematological malignancy that affects the lymphoid lineage precursor cells such as B and T cells, leading to an accumulation of lymphoblasts. It is the most common type of cancer in children, but it can also affect adults. ALL requires immediate treatment as its leukemogenesis is rapid and aggressive (Malard and Mohty, 2020). The clinical features of ALL can vary depending on several factors, including the age of the patient, the subtype of ALL and the stage of the disease at time of the diagnostic (Inaba and Mullighan, 2020; Malard and Mohty, 2020). Conventional diagnostics for the subtypes of ALL include immunophenotyping, cytogenetic and molecular analysis (Duffield et al., 2023).

The molecular features of ALL are complex and involve genetic alterations that disrupt normal cellular processes, including differentiation and proliferation. Some of the key molecular features of ALL include: a) Chromosomal abnormalities: the t(9; 22) translocation, which results in the fusion of the BCR and ABL1 genes, commonly known as Philadelphia chromosome-positive (Ph+) ALL. Several fusions affecting the MLL (or KMT2A) gene on chromosome 11q23, which also have important well established clinical implications. Patients harboring this translocation often have a poorer prognosis than those without MLL rearrangements. The translocation t(12; 21) (p13; q22) generates the fusion of ETV6-RUNX1 genes, which also carry significant clinical implications for patients (Bhojwani et al., 2015; Luca, 2021). b) Gene mutations: ALL is also characterized by mutations in genes that regulate cell growth, differentiation, and survival, such as TP53, KRAS, IKZF1 and NOTCH1. These mutations can contribute to disease progression and drug resistance (Inaba and Mullighan, 2020; Malard and Mohty, 2020; Chang et al., 2021). c) Aberrant gene expression resulting from genetic alterations, such as mutations, and epigenetic changes such as DNA methylation, histone modification, and dysregulated expression of non-coding RNAs. These alterations can affect the expression of genes involved in cell cycle regulation, DNA repair, and apoptosis, ultimately leading to uncontrolled cell growth and survival (Fleur-Lominy et al., 2020; Chang et al., 2021). d) Alterations in signaling pathways that regulate cell growth, survival, and differentiation, such as the JAK-STAT pathway, involved in cytokine signaling, which is frequently activated in ALL, leading to increased cell proliferation and survival (Sasaki et al., 2022b). Understanding these features at the molecular level is crucial for the development of targeted therapies that can precisely address the genetic alterations and signaling pathways responsible for driving disease progression.

The clustered regularly interspaced short palindromic repeats (CRISPR)/Cas9 is a technology for precise and efficient genome editing that has changed the field of biomedical research. The technique relies on just two exogenous elements: the Cas9 endonuclease and a single guide RNA (sgRNA), which is a fusion of crRNA and tracrRNA, resulting in a chimeric molecule performing both functions (Jinek et al., 2012; Zhang et al., 2014). CRISPR/Cas9 has significantly broadened the research landscape for comprehending and addressing ALL, as it provides the capability to precisely target genes directly associated with the disease. Here we discuss the current developments on CRISPR/Cas9-based genome editing used for the study and treatment of acute lymphoblastic leukemia and its perspectives. Our focus will encompass three key aspects: 1) Genetic target identification: identifying genes essential for cancer survival holds the potential to pave the way for the development of more effective targeted therapies and, ultimately, a potential cure. 2) Overcome drug resistance: CRISPR/Cas9 has the potential to enhance the effectiveness of existing treatment modalities. 3) Immunotherapy: leveraging CRISPR/Cas9 to modify a patient’s own T lymphocytes for precise targeting of cancer cells (González-Romero et al., 2019).

## 2 Genome editing in ALL with CRISPR/Cas9 system

Most of the recent studies on acute lymphoblastic leukemia (ALL) using CRISPR/Cas9 technology have focused on knocking out or knocking in genes, or conducting genome-wide screens to identify and validate therapeutic targets (Table 1). For instance, Jaiswal et al. conducted a CRISPR/Cas9 screening on MLL-rearranged B-ALL positive (SEM) and negative (Nalm-6) cell lines to identify dependencies on several genes, including *EIF3E, EPRS*, and *USO1*. To validate the screening results, a specific CRISPR/Cas9-mediated knockout of *USO1* was generated in *MLL-*translocated B-ALL cells. The *USO1* knockout reduced cell growth, promoted cell death and cell cycle arrest at G0/G1 phase. Further transcriptomic analysis of *USO1* knockout cells revealed alterations in pathways related to cellular proliferation, such as mTOR signaling, RNA metabolism, and targets of MYC (Jaiswal et al., 2021). This research led to the identification and validation of a novel target in MLL-rearranged leukemia subtype.

**TABLE 1:** Description of the studies using CRISPR/Cas9 in Acute Lymphoblastic Leukemia.

Leukemia type	Target	Type of study	Relevant findings	Reference
T-ALL	tRNA-Val (Valine transfer RNA)	*In vitro* (cell lines CUTLL1, Jurkat, DND41 and KOPTK1)	The expression of valine aminoacyl tRNA synthetase is transcriptionally upregulated by NOTCH1, a prominent oncogene in T-ALL. A genome-wide CRISPR–Cas9 loss-of-function screen performed under varying valine conditions identified several genes, such as SLC7A5 and BCL2, whose genetic deletion operated synergistically with valine restriction to decrease T-ALL growth.	Thandapani, P. 2022 (Thandapani et al., 2022)
T-ALL	LCK (lymphocyte-specific protein tyrosine kinase)	*In vitro* (cell lines HSB-2, PF-382, and SUP-T1)	CRISPR/Cas9 screen validated LCK dependency in T-ALL and providing evidence that LCK inhibition is the driver for Dasatinib sensitivity in this cancer.	Gocho, Y. **2021** (Gocho et al., 2021)
T-ALL	LDHA (lactate dehydrogenase A)	Zebrafish model	Inhibition of T-ALL progression was accomplished through targeting crucial regulators of tumorigenesis, including c-Myc. In zebrafish, the knockdown of the LDHA gene reduced T-ALL progression and significantly increased the number of apoptotic leukemic cells.	Yu, H. **2020** (Yu et al., 2020)
T-ALL B-ALL	RHEB (Ras homologue enriched in brain) PAX5 (Paired Box 5)	*In vitro* (cell lines Jurakt, HPB-ALL, PEER, MOLT-4, and SKW-3) *In vivo* *In vitro* (cell line ALL-697); Patient-derived pre-B ALL	RHEB is a protein that plays a role in regulating the cell cycle, specifically in normal hematopoiesis. The suppression of *de novo* nucleotide biosynthesis due to its loss leads to cell death *in vitro* and *in vivo*, which promotes human T-ALL. CRISPR-Cas9 screen identified PAX5 as a regulator of CD58 expression, an important effector in T cell immune response initiation. CD58-mediated CRISPR/Cas9 knockout in ALL cells *in vitro* failed to trigger T cell activation and proliferation in the presence of the immunotherapeutic agent blinatumomab, thus decreasing its sensitivity. This study highlights an important mechanism of immunotherapy resistance in ALL where PAX5 epigenetically orchestrates CD58 transcription and modulates blinatumomab response. In another study, CRISPR/Cas9 is used to knock out the PAX5 transcription factor, which is known to safeguard against leukemic transformation by limiting glucose and energy supply, in patient-derived pre-B ALL. This elimination of restriction increases glucose uptake and ATP levels by upregulating the expression of MYC, NR3C1, TXNIP, and CNR2, thereby contributing to leukemic transformation.	Pham, L.T. **2022** (Pham et al., 2022) Li, Y. **2022** (Li et al., 2022); Chan, L.N. **2017** (Chan et al., 2017)
Ph-like ALL	STATs, mTOR complex	*In vitro* (cell lines MUTZ5, KOPN49, and TVA1)	STATs are largely dispensable for the survival of Ph-like ALL cells. The depletion of either MTOR or RPTOR, which encode components of the mTORC1 complex, significantly altered cell fitness regardless of RAS mutation status. This suggests that the mTORC1 pathway plays a key role in the survival of Ph-like ALL cells. Treatment should be stratified according to co-occurring mutations.	Sasaki, K. **2022** (Sasaki et al., 2022a)
Ph-like ALL	Variant rs3824662 in *GATA3*	*In vitro* (cell line GM12878)	The genetic variant rs3824662 knock-in is associated with the upregulation of GATA3 transcriptional activation, which enhances the oncogenic effects of JAK-STAT during leukemogenesis. This germline noncoding variant contributes to the activation of oncogenes and epigenetic regulation.	Yang, H. **2022** (Yang et al., 2022)
B-ALL	USO1 (Vesicle transport factor)	*In vitro* (cell line Nalm-6)	USO1 knockout reduced cell growth, promoted cell death and cell cycle arrest at G0/G1 phase. Alterations in mTOR signaling pathway was also observed.	Jaiswal, A.K. **2021** (Jaiswal et al., 2021)
B-ALL	IKZF1 and IK6 (IKAROS family zinc finger 1; IKAROS isoform 6)	*In vitro* (cell line) Nalm-6	IKZF1-mutated B-ALL is aggressive, infiltrating and resistant. A viable approach is to generate reliable model systems by CRISPR engineering of human cell lines.	Rogers, J. **2021** (Rogers et al., 2021)
B-ALL	BTK and GCN2 (Bruton’s tyrosine kinase; General control nonderepressible-2)	*In vitro* (Nalm-6 cell line)	Knockout of BTK and GCN2 followed by ASNase (asparaginase) treatment promoted apoptosis *in vitro*. Moreover, pharmacological inhibition of BTK by ibrutinib could also suppress GCN2 function *in vitro* thorough suppression of c-MYC expression. This study revealed a mechanism of synergy between the kinase inhibitor ibrutinib and ASNase treatment that could benefit ALL patients.	Butler,M. **2021** (Butler et al., 2021)
B-ALL	USF2 (Upstream Transcriptin Factor 2)	*In vitro* (cell line SEM)	The functional assays showed that CRISPR/Cas9 mediated knockout for USF2 significantly downregulated HOXA9 expression and impaired cell proliferation *in vitro*, whereas ectopic expression of HOXA9 was able to rescue cell proliferation in USF2 knockout cells.	Zhang, H. **2020** (Zhang et al., 2020)

In another study, a genome-wide CRISPR/Cas9 screen identified PAX5 as a regulator of CD58 expression, an important effector in T cell immune response initiation. PAX5 is a transcription factor that plays a crucial role in B cell development and is part of a complex network of transcription factors, including IKZF1, E2A, EBF1, and RUNX1. Furthermore, CD58-mediated CRISPR/Cas9 knockout in ALL cells *in vitro* failed to trigger T cell activation and proliferation in the presence of the immunotherapeutic agent blinatumomab, thus decreasing its sensitivity (Li et al., 2022). This study highlights an important mechanism of immunotherapy resistance in ALL where PAX5 epigenetically orchestrates CD58 transcription and modulates blinatumomab response.

HOXA9 is a transcription factor overexpressed in MLL rearranged leukemias and is correlated with poor prognosis in ALL patients (Al-Kershi et al., 2019; Zhang et al., 2020). Dysregulated expression of HOXA9 plays an important role in leukemic transformation and leukemic cell proliferation. Using a knock-in strategy and a CRISPR/Cas9 screen to identify transcription factors regulating HOXA9 expression, two novel regulators were proposed: USF2 and DOT1L. Focusing on USF2 candidate, functional assays showed that its CRISPR/Cas9 mediated knockout significantly downregulated HOXA9 expression and impaired cell proliferation *in vitro*, whereas ectopic expression of HOXA9 was able to rescue cell proliferation in USF2 knockout cells (Zhang et al., 2020).

Another interesting study performed a CRISPR/Cas9 screening targeting protein kinases during asparaginase (ASNase) treatment to identify potential targets of the amino acid response pathway involved in ASNase sensitivity. Among several identified targets, BTK and GCN2 were enriched. Knockout of BTK and GCN2 followed by ASNase treatment promoted apoptosis *in vitro*. Moreover, the pharmacological inhibition of BTK by ibrutinib could also suppress GCN2 function *in vitro* thorough suppression of c-MYC expression (Butler et al., 2021). This study revealed a mechanism of synergy between the kinase inhibitor ibrutinib and ASNase treatment that could benefit ALL patients.

Normal cells are dependent on an adequate supply of energy to support their survival, and so are tumor cells. There disorders in abnormal lipid metabolism, amino acid metabolism, mitochondrial biogenesis, and other bioenergy metabolism pathways in cancer cells in addition to glucose metabolism. The disorder and metabolic reprogramming give support to the proliferation, migration, and invasion of cancer cells (Navarro et al., 2022). Therefore, understanding the energy metabolism mechanism might provide new targets in cancer treatment.

The aforementioned PAX5 transcription factor is known to safeguard leukemic transformation by limiting glucose and energy supply. CRISPR/Cas9 mediated knockout of PAX5 abrogates this restriction and increases glucose uptake and ATP levels, through the upregulation of MYC, NR3C1, TXNIP and CNR2 expression, all of them identified as central effectors of energy supply restriction in B cells and regulated by PAX5, and thus contributing to leukemic transformation (Chan et al., 2017).

A CRISPR/Cas9 screening focused on mTOR pathway effectors revealed that RHEB, a protein involved in cell cycle regulation, including normal hematopoiesis, could be a novel target in ALL. RHEB loss suppressed *de novo* nucleotide biosynthesis, leading to human T-ALL promoted cell death *in vitro* and *in vivo*, both in mouse and xenograft models (Pham et al., 2022). In addition, several other studies have used the CRISPR/Cas9 system to identify important genetic vulnerabilities in ALL, as summarized in Table 1.

## 3 CRISPR/Cas9 system to overcome drug resistance

The primary challenge in cancer treatment today is the ability of cancer cells to develop resistance to chemotherapeutic drugs. This resistance, which can be either inherent or acquired, is primarily driven by the dysregulation of genes related to chemoresistance. As such, understanding the molecular mechanisms that contribute to this resistance and identifying the genes involved are crucial. This knowledge could pave the way for the discovery of new therapeutic targets, the development of novel drugs, and the establishment of more effective combination treatment strategies to overcome this resistance. Cytarabine, also known as cytosine arabinoside (Ara-C) is a key drug used in the treatment of ALL. Ara-C, a deoxyadenosine analog, requires phosphorylation by deoxycytidine kinase (DCK) to transform into its active form, cytosine arabinoside triphosphate (Ara-CTP). An *in vitro* study demonstrated that higher DCK expression correlates with increased sensitivity to Ara-C. Furthermore, it was observed that DCK knockout via CRISPR/Cas9 induces chemoresistance to Ara-C, in contrast to clofarabine (a second-generation deoxyadenosine analog) (Huang et al., 2018). These findings highlight the role of DCK expression in Ara-C chemoresistance in ALL and suggest that clofarabine could be a more effective treatment option for ALL resistant to cytarabine.

Antimetabolites, such as methotrexate, are well-established in the treatment of ALL. Resistance to this treatment line poses a significant challenge to disease control (Wojtuszkiewicz et al., 2015). Yet, the mechanisms underlying such resistance remain largely unexplored. A study showed that low expression of ARID5B is associated with relapse in ALL patients, and that this could be due to a role of ARID5B in the process of chemotherapeutic resistance. A CRISPR/Cas9 mediated knockdown of ARID5B was achieved using a sgRNA targeting the suppressor protein KRAB to the ARID5B promoter suppressing its transcription. The suppression of ARID5B inhibited ALL cell proliferation, caused partial cell-cycle arrest and led to resistance specific to both antimetabolite drugs 6-mercaptopurine and methotrexate in ALL cells with ARID5B downregulation (Zhao et al., 2022). This data indicates that ARID5B expression could be a molecular biomarker of antimetabolite drug sensitivity in ALL.

ETV6/RUNX1 fusion oncogene commonly found in ALL was also successfully targeted using CRISPR/Cas9. In this case, guide RNA was designed to target the specific ETV6 and RUNX1 fusion region. Subsequent qPCR reported a reduction in the mRNA of ETV6/RUNX1 fusion construct following CRISPR/Cas9–mediated knockout of the fusion region. Moreover, functional studies of the ETV6/RUNX1 knockout cells showed a reduction in proliferative potential and an increased sensibility to treatment (Montaño et al., 2020). Since the tyrosine kinase inhibitors (TKIs) are the most efficient option to treat Ph + leukemia, and the resistances to TKIs are still a problem due to new acquired mutations in the BCR-ABL gene, genome editing could be a tool to overcome this issue. CRISPR/Cas9 was used in both *in vitro* and *in vivo* model of Ph + ALL harboring the T315I one-point mutation, with an intron-intron junction targeted strategy. Disruption of the expression of the fusion protein was observed, which resulted in a decrease in viability *in vitro*, and a progression of leukemia *in vitro* (Tan et al., 2020).

These studies highlight the importance of CRISPR/Cas9 as a novel tool to explore genetic vulnerabilities to overcome drug resistance in ALL treatment.

## 4 Clinical implications of CRISPR/Cas9 system

While chemotherapy and/or radiotherapy have traditionally been the standard treatments for Acute Lymphoblastic Leukemia (ALL), immunotherapies have recently emerged as a significant alternative treatment strategy. A prime example of this, is the use of Chimeric Antigen Receptors (CARs). These are proteins that have been genetically engineered to enable T cells (now referred to as CAR-T cells) to recognize a specific antigen present in tumor cells (Sheykhhasan et al., 2021). The CRISPR/Cas9 technology can be utilized to create allogeneic CAR-T cells that do not cause graft-versus-host disease (GVHD). This is achieved by disrupting proteins that are crucial for the recognition of the antigen-major histocompatibility complex (MHC), such as the T-cell receptor (TCR) beta chain and beta-2-microglobulin (B2M) (Dimitri et al., 2022).

The significant impact that CRISPR/Cas9 technology has on current clinical practice is undeniable, including in the creation of CAR-T cells for the treatment of ALL. Numerous clinical trials are currently underway to further explore the potential of these CRISPR/Cas9-generated CAR-T cells in ALL treatment (Table 2).

**TABLE 2:** Ongoing clinical trials with CRISPR/Cas9-engineered CAR-T cells.

ClinicalTrials.gov Identifier	CAR-T name	Condition or disease	Location	Recruitment status	Last update posted
NCT04037566	XYF19	Acute Lymphoblastic Leukemia (ALL) in Relapse; Refractory Acute Lymphoblastic Leukemia (All); B-Cell Lymphoma	China	Recruiting	30 July 2019
NCT04035434	CTX110	Non-Hodgkin Lymphoma; B-cell Lymphoma; Adult B-cell ALL	Multicenter: United States, Australia, Canada, Germany, Spain	Recruiting	9 May 2022
NCT05397184	TvT CAR7	Relapsed/Refractor T-cell Acute Lymphoid Leukemia	United Kingdom	Recruiting	31 May 2022

A phase 1, open-label, non-randomized clinical trial treated six children with relapsed/refractory CD19-positive B cell acute lymphoblastic leukemia (B-ALL) (NCT04557436). Four of six patients infused with TT52CAR19 T cells exhibited cell expansion, achieved remission, and then proceeded to receive allogeneic stem cell transplantation. Cytokine release syndrome occurred in two patients, transient neurotoxicity occurred in one patient, and one patient developed skin GVHD (Ottaviano et al., 2022).

An open-label, phase I study with a dose-escalation treated six relapsed/refractory B-cell acute lymphoblastic leukemia patients with CTA101 cell (NCT04227015). Patients received infusions at doses of 1×10^6^ CAR + T cells/kg body weight (3 patients) and 3×10^6^ CAR + T cells/kg body weight (3 patients). Cytokine release syndrome occurred in all patients. No dose-limiting toxicity, GVHD, neurotoxicity, or genome editing-associated adverse events were observed. After 28 days of CTA101 infusion, the complete remission (CR) rate was 83.3%. With a median follow-up of 4.3 months, 3 of the 5 patients who achieved complete remission (CR) or CR with incomplete hematologic recovery (CR/CRi) remained minimal residual disease (MRD) negative (Hu et al., 2020).

Both studies provided evidence of the therapeutic feasibility, safety and potential of CRISPR/Cas9-engineered cell therapy in ALL.

## 5 Challenges and future directions

Translating genome editing technologies to the clinical setting requires two main concerns to be addressed: the safety and efficacy of treatments. The off-target effect remains one of the major obstacles of this technology. The unexpected off-target mutagenesis may cause the ablation of tumor suppressor genes or the activation of oncogenes and its pathways, therefore increasing the clinical risks of CRISPR/Cas9 based therapy (Naeem et al., 2020).

Minimizing and controlling the off-target effects of genome editing has become a critical concern. Among the strategies developed, the use of paired Cas9 nickases induce double-stranded cleavage and require two sgRNAs. This approach significantly increases specificity, as it is highly improbable for an off-target sequence to be complementary to both sgRNAs (Mali et al., 2013; Ran et al., 2013). Furthermore, variants of the commonly used protein, *Streptococcus pyogenes* Cas9 (SpCas9), such as SpCas9-HF1 (*Streptococcus pyogenes* Cas9 High Fidelity 1), have been developed. These variants leverage the surplus energy from the DNA/SpCas9 interaction to recognize sequences that are not perfectly complementary to the sgRNA. Additionally, eSpCas9 (enhanced specificity SpCas9) was created based on the identification of specific DNA/SpCas9 interaction regions that preferentially interact with non-specific targets. The molecular composition of the DNA/SpCas9 complex was used in the production of these variants, resulting in a decrease in non-specific bindings with their target DNA site (Slaymaker et al., 2013; Kleinstiver et al., 2016). Future research will be essential for enhancing our existing genetic tools, spanning from *in silico* analysis to wet lab strategies. This will aim to mitigate off-target effects and boost gene editing efficiency.

Another challenge is the immune response of eukaryotic cells to Cas9, triggered by antibodies against *Streptococcus pyogenes* and *Staphylococcus aureus*. This immune response could potentially affect the efficiency of Cas9 activity and should be taken into account when developing personalized immunotherapies. However, this issue could be circumvented by using alternative nucleases such as Cas12 or Cas13, thereby eliminating the need for immunosuppressive medications (Ewaisha and Anderson, 2023).

Furthermore, the evidence demonstrating the substantial clinical potential of utilizing the CRISPR/Cas9 tool in treating ALL and other diseases further confirms the indisputable efficacy of CRISPR/Cas9 with targets already under clinical investigation. For instance, in a phase I study aimed at modifying allogeneic pancreatic endoderm cells using CRISPR/Cas9, research efforts are currently applied in treating type I diabetes, an autoimmune disease (Lim and Kim, 2022; CRISPR Theraputics, 2023). Other phase I clinical trials are ongoing in HIV infection for gene editing of the CCR5 receptor, inhibition of viral replication, and HIV resistance (Li et al., 2015; Xu et al., 2017). Sickle cell disease targets correction of the *β-globin* subunit gene in hemoglobin via CRISPR/Cas9-based genome editing in Phase I and II of clinical trials, with modified hematopoietic stem cells and modified CD34^+^ hematopoietic stem cells (Frangoul et al., 2021; Gillmore et al., 2021).

Another application of CRISPR with great potential to reach clinical trials in the future, is the prime editing technique. Prime editing, which has a few advantages over other CRISPR/Cas9-mediated genome-editing methods, has been swiftly implemented in in vivo studies due to its potential to treat a variety of genetic disorders. This method combines a disabled Cas9 protein, capable of nicking DNA, with a modified prime editing guide RNA (pegRNA). The pegRNA not only specifies the location of the edit, but also carries new genetic instructions to be installed at that location (Zhao et al., 2023). Although prime editing has allowed specific gene modification in various cell types (Kim et al., 2021), its low editing efficiency (Ravichandran and Maddalo, 2023a) and the lack of an efficient *in vivo* delivery system remains crucial challenges for its implementation (Zhao et al., 2023).

Finally, the use of CRISPR has facilitated the identification of critical targets for leukemogenesis that can guide research in the development of new therapeutic approaches (Akram et al., 2022). The CRISPR activation or inhibition systems (CRISPRa/i) have accelerated the discovery of genes linked to cellular reprogramming by rapidly identifying genetic targets (Ravichandran and Maddalo, 2023b). CRISPRa is a modified version of CRISPR that utilizes a catalytically inactive (d) Cas9 fused with a transcriptional effector to activate target gene expression. On the other hand, CRISPRi involves guide RNA and a transcriptional effector arm which, when navigating to a target genome locus, represses gene expression downstream instead of activating it. While these systems have demonstrated their versatility in identifying potential new targets, it is crucial that these targets undergo rigorous validation using orthogonal approaches to be able to reach the clinical stage.

The CRISPR/Cas9 system has already showcased its potential in studying the molecular biology and pathogenesis of genetic aberrations in Acute Lymphoblastic Leukemia (ALL), both *in vitro* and *in vivo*. This has enabled a deeper understanding of the disease’s genomics, phenotypes, and therapeutic targets. Consequently, it has broadened the scope for functional genomics studies and fostered the advancement of personalized medicine options for ALL in the near future.
